# Natural Reading in Parkinson’s Disease With and Without Mild Cognitive Impairment

**DOI:** 10.3389/fnagi.2020.00120

**Published:** 2020-05-22

**Authors:** Lena Stock, Charlotte Krüger-Zechlin, Zain Deeb, Lars Timmermann, Josefine Waldthaler

**Affiliations:** ^1^Department of Neurology, University Hospital Marburg, Marburg, Germany; ^2^Center for Mind, Brain and Behavior - CMBB, Universities Marburg and Gießen, Marburg, Germany

**Keywords:** Parkinson’s disease, reading, eye movements, eye tracking, cognition, mild cognitive impairment

## Abstract

**Background:** Patients with Parkinson’s disease (PD) show eye movement abnormalities and frequently complain about difficulties in reading. So far, it is unclear whether basal ganglia dysfunction or cognitive impairment has a greater impact on eye movements during reading.

**Objective:** To analyze eye movement behavior during a natural reading task with respect to cognitive state and dopaminergic therapy in PD and healthy controls.

**Methods:** Eye movements of 59 PD patients and 29 age- and education-matched healthy controls were recorded during mute, self-paced reading of a text. 25 cognitively normal PD patients performed the task additionally in off medication state. Clinical assessment included a comprehensive neuropsychological test battery and the motor section of MDS—Unified Parkinson’s Disease Rating Scale (MDS-UPDRS).

**Results:** PD-mild cognitive impairment (MCI) was diagnosed in 21 patients. Reading speed was significantly reduced in PD-MCI compared to healthy controls and PD patients without MCI due to higher numbers of progressive saccades. Cognitively intact PD patients showed no significant alterations of reading speed or eye movement pattern during reading. The fixation duration tended to be prolonged in PD compared to healthy controls and decreased significantly after levodopa intake. Scores for executive functions, attention, and language correlated with reading speed in the PD group.

**Conclusion:** The present study is the first to reveal (1) reduced reading speed with altered reading pattern in PD with MCI and (2) a relevant impact of levodopa on fixation duration during reading in PD. Further research is needed to determine whether therapeutic interventions, e.g., levodopa or neuropsychological training, improve the subjective reading experience for patients with PD.

## Introduction

Basic oculomotor performance is impaired in patients with Parkinson’s disease (PD), as demonstrated in a large number of studies on reflexive and voluntary saccades (Rascol et al., 1989; Michell et al., 2006; MacAskill et al., 2012). Generally speaking, voluntary saccades are hypometric and tend to be executed with a prolonged latency in PD. The impaired oculomotor control may also affect reading performance. Eye movements during natural reading, resembling activities like reading a book or newspaper, are not a well investigated area in PD, despite the possible impact on daily life. In a recent study, 42% of PD patients complained about reading difficulties which many of them traced back to impaired vision or concentration (Davidsdottir et al., 2005). First evidence for impaired eye movements during reading in an otherwise cognitively intact PD patient was provided in a case report in 2016 (Yu et al., 2016).

From an eye movement perspective, reading is a series of fixations and saccades. The purpose of performing saccades during reading is to bring new words onto the fovea while the containing information is gathered and processed mainly during fixations (Rayner et al., 1998). Regressions are saccades that are made in the opposite direction of reading. Whenever a reader experiences problems in processing a word during a fixation, short regressive saccades within this word are made. Longer regressions of 10 letters or more may more likely indicate difficulties with integrating a word into the meaning of a sentence or with understanding the text as a whole (Rayner et al., 1998). While eye movement patterns differ as a function of several linguistic properties (Clifton et al., 2007), the perception of whether a word or a text is difficult or not is obviously largely dependent on the level of individual reading skill. In general, reading skills can be defined either by reading speed or by reading comprehension, whereby reading speed is the more reliable predictor for eye movement patterns (Krieber et al., 2016).

In a small pilot study of 19 PD patients, we demonstrated reduced reading speed in natural reading, caused by an increased number of regressions and longer fixation durations. The increased fixation duration correlated with MoCA score (Waldthaler et al., 2018). Thus, we hypothesized that the reading pattern of PD patients would differ from healthy individuals, but that slower reading speed would rather be caused by cognitive deficits than by the motor impairment. Therefore, the objectives of the current study were to explore the relationship of cognitive impairment and reading behavior in PD. Furthermore, we aimed to study the effect of levodopa on eye movements during reading.

## Materials and Methods

The study was approved by the local ethical committee at the University Hospital Marburg and was conducted in accordance with the Declaration of Helsinki. All participants gave written informed consent.

59 patients diagnosed with PD according to UK Brain Bank Criteria for a diagnosis of PD and 29 age-, sex-, as well as education-matched healthy control subjects with normal or corrected-to-normal visual acuity were recruited for this study. All participants were native German speakers. Subjects with other relevant ophthalmological, neurological, neurodegenerative, or psychiatric disorders, in particular a current diagnosis of depression, which result in reduced visual acuity or might affect oculomotor behavior were excluded.

Parkinson’s disease patients performed the reading task after intake of their regular dose of medication. To study the impact of levodopa on reading speed and eye movements during reading, 25 PD patients with normal cognitive functions completed the reading task additionally in off medication state, e.g., after a 12-h withdrawal of dopaminergic therapy.

### Motor and Cognitive Assessments

Motor performance was assessed using the motor section (part 3) of the MDS—Unified Parkinson’s disease Rating Scale (MDS-UPDRS) (Goetz et al., 2007). Cognitive testing was performed in on medication state. In addition to Montreal Cognitive Assessment (MoCA), each subject underwent a comprehensive neuropsychological test battery. According to the recommendations of the MDS Task Force Criteria for the diagnosis of mild cognitive impairment (MCI), two tests for each of the five cognitive domains attention (Trail Making Test A, forward digit span), executive functions (Trail Making Test B, Frontal Assessment Battery), language (semantic and phonemic verbal fluency in the “supermarket” task and words beginning with the letters “F” and “S”), memory (immediate and delayed recall of the 10-item list included in Demtect and recall of Rey Complex Figure), and visual-spatial function (clock drawing test and Rey Complex Figure copying) were included (Goldman et al., 2015). A performance below two standard deviations (SDs) compared to age-matched reference groups in the literature was defined as pathological (Caffarra et al., 2002; Kalbe et al., 2005; Benke et al., 2013; Larouche et al., 2016; St-Hilaire et al., 2018).

### Eye Tracking Data Acquisition

The eye tracking session took place in a dimly lit room and stimuli were presented on a computer monitor in a viewing distance of 60 cm using the open-source program OpenSesame (Mathôt et al., 2012). Participants were sitting in a comfortable chair with their head stabilized by a chin rest and a forehead bar. A video-based eye tracker (EyeLink 1000 PLUS, SR Research Ltd.) which is an established measuring tool in oculomotor research recorded the monocular position of the right eye with a sampling rate of 500 Hz. After calibration with a standard five-point grid, participants silently read one of two German texts consisting of 129, respectively, 134 words in black, regular font Times New Roman with a letter size of 12 point on white, illuminated background. The specific text size and viewing distance had been selected to simulate realistic conditions of reading a newspaper article on an A5 page. The participants were requested to read the text as per how they would, with any visual aids if necessary.

To be able to compare the reading results between the two study visits, two texts with the same category (animals) and difficulty were chosen from the International reading speed texts (IReST) (Trauzettel-Klosinski and Dietz, 2012). The IReST texts are available in different languages and are matched for content, length, and linguistic complexity for repeated measurements.

The sessions in on and off medication state were implemented in an alternating order to prevent potential systematic error due to learning effects. Healthy controls performed the reading task only once, whereby the two texts were alternated from subject to subject.

### Eye Tracking Data Analysis

A parsing system is incorporated in the EyeLink 1000 PLUS that analyses eye position data into saccades, fixations, and blinks. This raw dataset was analyzed line by line in R using the eyelinker package (Se, 1993; Team and Development Core Team, 2016). Thresholds of 40°/s peak velocity for saccade detection and 60 ms for minimum fixation duration were used. Reading speed, i.e., words per minute (wpm), was calculated as the time span between the first fixation on the first word and the last fixation on the last word of the text. Additionally, numbers (per word) and amplitudes of progressive and regressive saccades as well as fixation durations were calculated and extracted from the datasets for further statistical analysis.

### Statistical Analysis

Statistics were executed in Prism 8 (GraphPad Software, LLC). Normal distribution of data was tested with Shapiro–Wilk test. If data did not pass normality test, non-parametric tests were applied.

In the first part of the analysis, one-way ANOVA, respectively, Kruskal–Wallis test, followed by multiple comparisons with Dunn’s correction were performed to compare oculomotor outcomes of healthy controls, PD with normal cognition, and PD-MCI groups, as well as oculomotor outcomes between the three groups. Pearson’s coefficients were calculated to test for linear correlations between clinical data, cognitive scores, and oculomotor outcomes of reading. For correction for multiple testing, the false discovery rate method was used.

In the second part of the analysis, within-subject comparison of levodopa treatment effect was tested with paired *t*-test or Wilcoxon paired-rank test in the subgroup of 25 PD patients who performed the reading task in both on and off medication state.

All reported *P*-values are two-tailed and a *P*-value < 0.05 was considered significant.

## Results

### Neuropsychological Outcomes

Mild cognitive impairment was diagnosed in 21 PD patients (PD-MCI). Hence, 38 PD patients and all 29 healthy controls were declared as cognitively normal (PD-CN). The three groups did not differ in age, or years of education (Table 1). There were no differences in disease duration and levodopa-equivalent daily dosage (LEDD) in the PD groups. However, MDS-UPDRS in on medication state was slightly higher in the PD-MCI than in PD-CN group.

**TABLE 1:** Clinical characteristics and results of healthy controls (HC), cognitively normal PD patients (PD-CN), and PD-MCI patients.

	HC (*n* = 29)			PD-CN (*n* = 38)			PD-MCI (*n* = 21)			HC vs. PD-CN vs. PD-MCI	
	Mean	SD	*n* < 2 SD	Mean	SD	*n* < 2 SD	Mean	SD	*n* < 2SD	*p*	Multiple comparison
Gender (male/female)	18/11			30/8			15/6				HC vs. PD *P* = 0,2 PD-CN vs. PD-MCI *P* = 0,5
Age (years)	61.4	11.5		62.9	7.4		64.9	10.3		0.5^*a*^	
Years of education	13.2	2.3		13.5	1.9		13.3	1.8		0.7^*a*^	
Disease duration (years)	–	–		7.9	6.4		9.0	4.5		0.8^*t*^	
Levodopa equivalent daily dose (mg per day)	–	–		681.4	422.5		767.3	280.7		0.4^*t*^	
MDS-UPDRS III	–	–		25.5	12.5		33.4	12.9		0.05^*t*^	
MoCA	26.7	2.4		26.7	3.0		22.0	2.5		<0.0001^*k*^	HCvs. PD-CN *P* > 0.9 **HC vs. PD-MCI *P* < 0.0001 PD-CN vs. PD-MCI *P* < 0.0001**
Trail Making Test A (s)	31.9	10.0	0	36.9	10.1	2	69.3	45.3	9	0.0004	HC vs. PD-CN *P* = 0.4 **HC vs. PD-MCI *P* = 0,0002 PD-CN vs. PD-MCI *P* = 0,02**
Forward digit span (*n*)	4.8	1.0	0	4.5	1.3	0	3.9	1.7	4	0.3	
Trail Making Test B (s)	66.3	30.1	0	85.4	34.9	5	176.0	102.0	21	<0.0001	HCvs. PD-CN *P* = 0,08 **HC vs. PD-MCI *P* < 0.0001 PD-CN vs. PD-MCI *P* = 0,01**
Frontal Assessment Battery FAB (score, max. 18)	17.0	1.5	1	17.1	1.0	2	13.2	3.2	12	<0.0001	HCvs. PD-CN *P* > 0.9999 **HC vs. PD-MCI *P* < 0.0001 PD-CN vs. PD-MCI *P* < 0.0001**
Phonematic verbal fluency (*n*; “F”)	12.9	3.5	1	13.0	5.3	1	10.5	4.0	5	0.2	
Semantic verbal fluency (*n*; “supermarket”)	26.1	4.8	0	23.5	5.1	0	18.6	5.	2	<0.0001	HCvs. PD-CN *P* = 0.2 **HC vs. PD-MCI *P* < 0.0001 PD-CN vs. PD-MCI *P* = 0,01**
10-item word list (immediate and delayed recall) (*n*, max. 20)	13.4	4.2	1	13.1	4.2	2	9.6	4.0	6	0.01	HC vs. PD-CN *P* > 0.9 **HC vs. PD-MCI *P* = 0,02 PD-CN vs. PD-MCI *P* = 0,03**
RCF copying (score, max. 36)	34.3	2.5	0	34.1	11.8	0	26.	19.5	0	0.0004	HC vs. PD-CN *P* = 0.2 **HC vs. PD-MCI *P* = 0.0002 PD-CN vs. PD-MCI *P* = 0,04**
RCF delayed recall (score, max. 36)	18.6	7.1	0	15.6	6.3	1	9.3	7.5	8	0.0002	HC vs. PD-CN *P* = 0.4 **HC vs. PD-MCI *P* < 0,0001 PD-CN vs. PD-MCI *P* = 0,007**
Clack drawing (score, max. 6)	1.2	0.6	1	1.2	0.6	0	2.3	1.2	10	<0.0001	HCvs. PD-CN *P* > 0.9 **HC vs. PD-MCI *P* < 0.0001 PD-CN vs. PD-MCI *P* < 0.0001**
Words per minute	231.5	53.3		230.7	53.7		176.1	42.3		0.0007^*k*^	HC vs. PD-CN *P* > 0.9 **HC vs. PD-MCI *P* = 0.003 PD-CN vs. PD-MCI *P* = 0.001**
Mean fixation duration	206.8	28.2		220.6	33.7		228.6	34.7		0.06^*a*^	
Mean amplitude progressive saccades	3.2	0.58		3.37	0.77		3.10	0.96		0.3^*k*^	
Mean amplitude regressive saccades	1.66	0.46		1.98	0.75		2.14	1.13		0.2^*k*^	
Progressive saccades per word	0.77	0.14		0.75	0.17		0.93	0.31		0.02^*a*^	HC vs. PD-CN *P* > 0.9 HC vs. PD-MCI *P* = 0.1 **PD-CN vs. PD-MCI *P* = 0.03**
Regressive saccades per word	0.14	0.08		0.15	0.09		0.25	0.19		0.1^*k*^	

*t = unpaired t test; a = one-way ANOVA; k = Kruskal Wallis test.*

Patients with PD-MCI performed significantly lower in almost all neuropsychological tests than PD-CN and healthy individuals, except for phonetic verbal fluency and forward digit spans (Table 2). There were no significant differences between PD-CN and healthy controls in the results of the cognitive tests.

**TABLE 2:** Results of PD patients with intact cognitive functions in off versus on medication state.

	PD-CN off (*n* = 25)		PD-CN on (*n* = 25)		HC vs. PD-CN off	PD-CN off vs. on
	Mean	SD	Mean	SD	*p*	
Words per minute	230	48	237	53	0.9^t^	0.4^t^
Mean fixation duration (ms)	221	28	212	27	0.07^t^	0.02^t^
Mean amplitude of progressive saccades (°)	3.21	0.59	3.14	0.52	0.7^t^	0.6^t^
Mean amplitude of regressive saccades (°)	1.83	0.59	1.88	0.72	0.06^t^	0.8^t^
Progressive saccades per word	0.73	0.12	0.72	0.13	0.2^t^	0.6^t^
Regressive saccades per word	0.14	0.10	0.15	0.10	0.9^t^	0.3^w^

*t = unpaired t test; a = one-way ANOVA; k = Kruskal Wallis test.*

### Effect of Cognitive Impairment on Reading

First, we explored which oculomotor parameters had the highest impact on overall reading speed. Here, reading speed was mainly determined by the numbers of progressive (*r* = −0.73, *p* < 0.0001) and regressive saccades (*r* = −0.60, *p* < 0.0001), to less extend by fixation duration (*r* = −0.36, *p* = 0.006).

**FIGURE 1:**
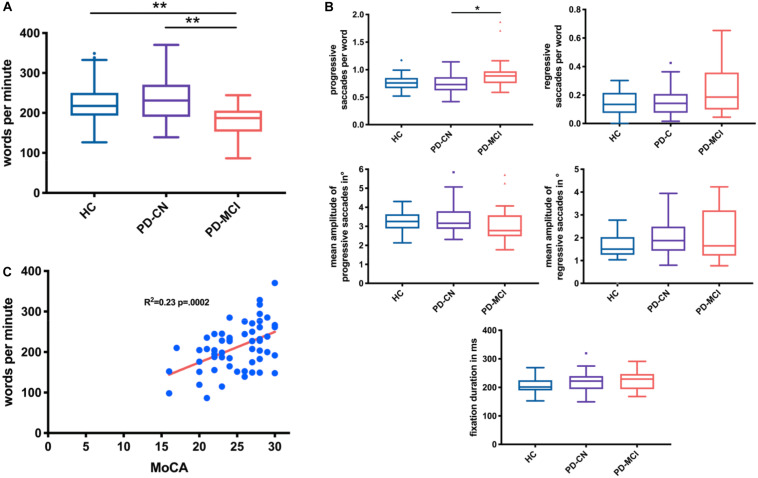
Box plots (Tukey) comparing reading speed **(A)** and reading measures **(B)** in healthy controls (HC), cognitively normal PD patients (PD-CN), and patients with PD-MCI. The total MoCA score correlated with reading speed (*P* = 0.0002, *r* = 0.48) **(C)** and with numbers of regressive and progressive saccades per word (*P* = 0.0005, *r* = 0.45; not shown). *p < 0.05, **p < 0.01.

**FIGURE 2:**
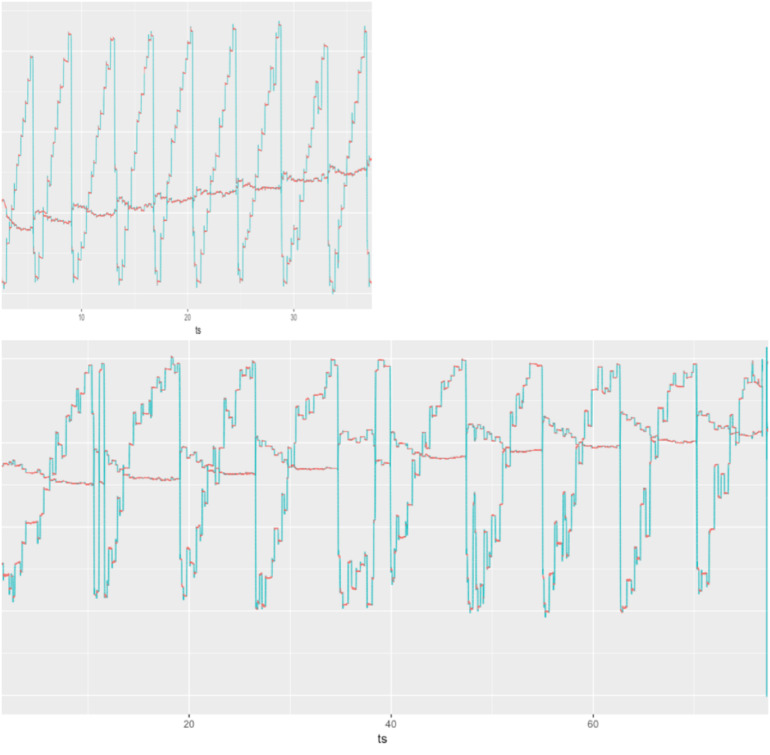
Raw eye movement data of exemplary reading patterns of a PD-CN patient (top) and a PD-MCI patient (bottom) to illustrate slower reading speed, higher numbers of saccades, and frequent disruptions with long regressive saccades in the patient with PD-MCI.

The three-group comparison of healthy controls, PD-CN, and PD-MCI resulted in an overall significant difference in wpm and number of progressive saccades per word as well as a trend for fixation duration (Table 1 and Figure 1). Multiple comparisons revealed that reading speed was reduced in PD-MCI compared to both, healthy controls and PD-CN. The number of progressive saccades per word was increased in PD-MCI compared to PD-CN (Table 1). Additionally, the distribution of numbers and amplitudes of regressive saccades were wider spread in the PD-MCI group than in healthy controls and PD-CN when compared visually (Figure 1). See also Figure 2 for an exemplary illustration of the differences in reading patterns.

**FIGURE 3:**
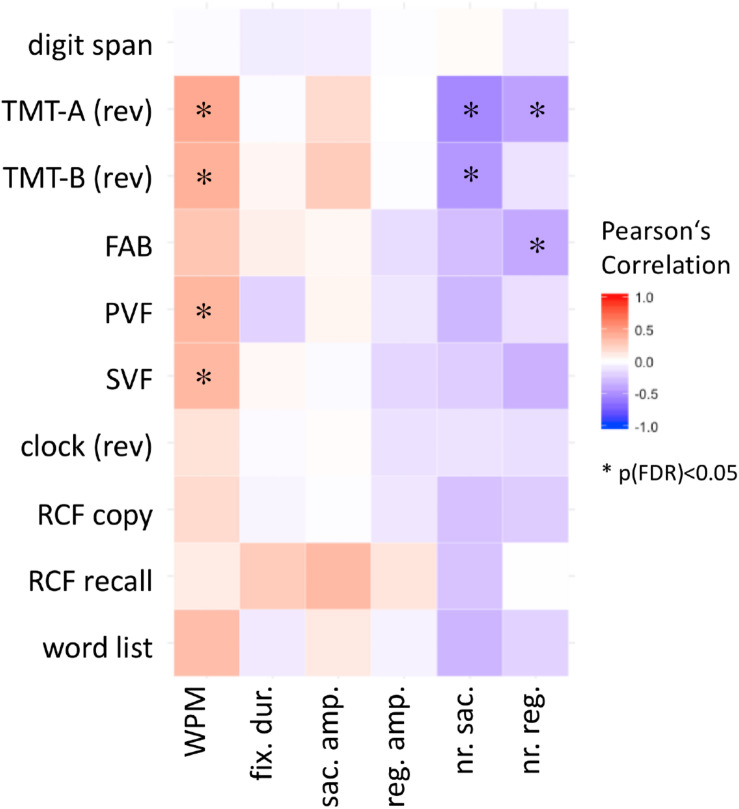
Correlations of reading parameters with cognitive tasks: heatmap displaying the Pearson correlation coefficients by color, asterisk marks significant correlation after FDR correction for multiple testing.

The results of multiple linear regressions of the neuropsychological scores of the PD groups with eye movement parameters are shown as a heatmap in Figure 3. After correction for multiple testing, correlations of reading speed remained significant with scores for executive functions, attention (TMT-A: *r* = 0.43, *P* = 0.001, *P*_*FDR*_ = 0.02; TMT-B: *r* = 0.40, *P* = 0.002, *P*_*FDR*_ = 0.03), and language (semantic verbal fluency: *r* = 0.36, *P* = 0.006, *P*_*FDR*_ = 0.04; phonetic verbal fluency: *r* = 0.37, *P* = 0.004, *P*_*FDR*_ = 0.03).

Further, numbers of progressive and regressive saccades per word correlated with the result of TMT-A (*r* = −0.54, *P* < 0.0001, *P*_*FDR*_ = 0.006) and TMT-B (*r* = −0.46, *P* = 0.0003, *P*_*FDR*_ = 0.009), respectively, TMT-A (*r* = −0.42, *P* = 0.002; *P*_*FDR*_ = 0.03) and FAB (*r* = −0.38, *P* = 0.003, *P*_*FDR*_ = 0.03). No correlations of reading parameters with age, sex, disease duration, LEDD, MDS-UPDRS (in on) or years of education were found (please see Supplementary Material S1).

### Medication Effect on Reading in Cognitively Intact PD

**FIGURE 4:**
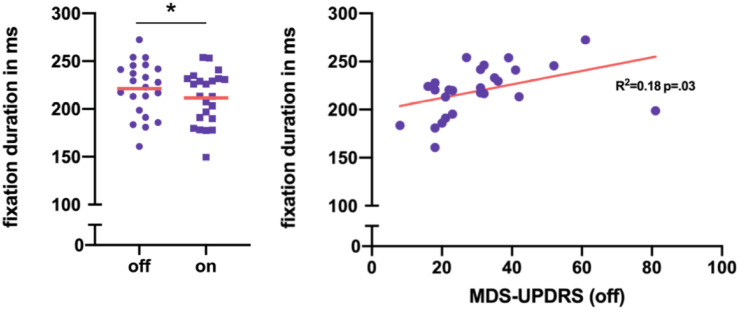
**(A)** Within-subject difference in mean fixation duration per PD-CN patients in on and off medication state. In total, the decrease of fixation duration after levodopa intake was significant. **(B)** Mean fixation duration showed a trend toward correlation with motor performance in MDS-UPDRS III in PD-CN. *p < 0.05.

Within-subject comparison of 25 PD patients with normal cognition in off versus on medication state revealed that levodopa intake lead to a significant decrease of fixation duration (Table 2 and Figure 4). All other reading parameters remained unchanged by levodopa.

In off medication state, solely mean fixation duration tended to be prolonged in PD patients compared to healthy individuals (Table 2). However, no significant differences in reading speed or any other oculomotor outcome of reading were found between healthy controls in cognitively normal PD patients in off medication state.

## Discussion

This study is the first to provide a comprehensive insight into the impact of cognitive state and the effect of levodopa on the eye movement pattern during natural reading in PD. The main results are that, while reading speed and patterns of cognitively normal PD patients did not differ from healthy individuals in on medication state, PD-MCI patients showed a reduction of reading speed due to higher numbers of saccades per word which was strongly related to neuropsychological deficits in executive function, attention, and language.

### The Influence of Levodopa on Reading Pattern in PD

The control of a sophisticated oculomotor task such as reading involves several cortical and subcortical brain regions (Bonhage et al., 2015). The frontal eye field (FEF), supplementary eye field (SEF), and intraparietal sulcus (IPS) have been identified as main regions related to cortical oculomotor control. Additionally, reading activates a well-established language network, involving the superior and middle temporal gyrus, angular gyrus, as well as the middle and inferior frontal gyrus (Choi et al., 2014). The mean fixation duration during reading is an indicator for the oculomotor, attentional, and language processing load that is necessary for understanding the text and planning the next saccade (Rayner and McConkie, 1976). Although the prolongation of mean fixation durations in cognitively normal PD patients did not reach significance in this cohort, the decrease after levodopa intake is still suggestive for a role of dopamine-dependent pathways in the oculomotor control of reading in PD. fMRI studies in healthy individuals demonstrated that longer fixation durations are mainly related to higher activity in SEF, middle, and superior temporal gyrus as well as lower activity in middle frontal gyrus and parietal regions (Henderson et al., 2015). Since studies using several functional imaging modalities showed that levodopa administration increases the striatal–frontal connectivity between the caudate nucleus and prefrontal cortex (Jahanshahi et al., 2010), the reduction of fixation duration after levodopa intake may result from a more effective processing due to improved connectivity between basal ganglia and prefrontal areas, respectively, superior colliculus.

Contrary to expectations, reduced saccade amplitudes, which is a consistent finding of reflexive and voluntary experimental saccade tasks, was not evident during reading. A reason might be that the top-down cognitive control mechanisms may be a stronger driver for saccade amplitudes in reading than in saccades to other cues.

### Changes in Reading Pattern With PD-MCI

Parkinson’s disease-MCI was associated with a reduced reading speed, mainly due to more saccades per word. The alterations of the reading pattern were related to performance in cognitive tasks assessing executive functions and attention.

Most research on the impact of cognitive domains on eye movements during reading was conducted in the field of developmental dyslexia. Reduced reading comprehension was found to be related to executive functions in both, dyslexic and healthy young readers (Locascio et al., 2010; Georgiou and Das, 2016). Thus, the predominant executive dysfunction in PD may result in a higher susceptibility for early reading impairment compared to neurodegenerative disorders with other cognitive profiles.

Regarding orthographic consistency, German is a transparent language, e.g., the graphem–phonem correspondence is highly consistent. In German readers, faster reading speed is associated with smaller numbers of fixations per word and total number of saccades as well as larger saccade amplitudes (Krieber et al., 2016). In general, readers of transparent languages tend to rely on sublexical rather than lexical decoding: they use bottom-up processing with careful first pass reading of smaller units and less re-reading. In comparison, readers of deep languages, like English, rather use top-down processing of larger units with more regressions and word skipping (Rau et al., 2015).

The higher the experience in a transparent language, the more the reader may also rely on a lexical access to word recognition. Vice versa, dyslexic readers use sublexical routes more extensively than normal readers (Wimmer, 1993). Although the texts used in this study were rather easy, the cognitive deficits in PD-MCI seem to lead to difficulties in understanding the text. PD-MCI readers then come back to serial sublexical decoding of the text, reflected by more saccades per word.

When cognitive deficits are present or the text is more difficult, the compensatory mechanism of increasing the number of saccades may not be sufficient. For instance, a Spanish study (orthographic consistency of Spanish is comparable to German) in Alzheimer’s disease demonstrated that patients performed a remarkably increased number of progressive and regressive saccades as well as longer fixation durations in spite of a reduced amplitude of saccades (Fernández et al., 2013). Even though numbers and amplitude of regressions did not differ between the groups in the current study, they showed a markedly wider spread distribution in individuals with PD-MCI. This may reflect greater difficulties in understanding the text during the first pass or problems with spatially planning of the specific target of a regression in PD-MCI patients.

In a smaller pilot study, which included 19 Swedish speaking patients, cognitive impairment assessed using the MoCA score was associated with longer fixation durations. This was not replicable in the current German-speaking cohort, although matching texts from the IReST set were used (Waldthaler et al., 2018). Linguistic studies showed that cross-linguistic processing differences while reading, which are most pronounced in children, persist in adult readers (Rau et al., 2015). Thus, Swedish and German readers may use different linguistic approaches to maintain comprehension of a text in spite of cognitive deficits. Swedish is considered to be orthographically deeper than German (Seymour et al., 2003). Therefore, we hypothesize that the primary compensatory mechanism in Swedish PD patients may differ, as those readers with lower MoCA scores tended to increase the fixation duration instead of executing more saccades (Waldthaler et al., 2018).

### Limitations and Clinical Implications

This study provides first evidence for a beneficial effect of medication on the natural reading pattern in PD as levodopa intake decreased fixation durations. Hereby, dopaminergic therapy may be efficious in improving a subjectively impaired reading experience in the earlier stages of PD. However, we cannot draw clear conclusions on the clinical impact since we did not assess the subjective impairment of reading. Another limitation is that no verbal comprehension or naming test was included in the neuropsychological test battery which would have been potentially been more useful to assess the language domain instead of the chosen phonemic and semantic fluency tests, because of the strong relationship among these tasks. Furthermore, a formal verbal comprehension test would have been particularly to further explore the relation between prolonged fixation duration and problems with reading comprehension.

A few additional limitations need to be discussed: First, the sample size of the PD-MCI group was relatively small, and these patients showed a slightly higher burden of motor symptoms than PD-CN patients which may have influenced the results. Furthermore, the control group, consisting of 29 healthy individuals, should be increased in future studies.

As discussed above, the results of the current study, especially regarding the alterations in reading pattern in PD-MCI, may be limited to German or languages with similar orthographic consistency. Furthermore, it is of notice that the identified reading pattern may not be specific for individuals with PD since investigations in Alzheimer’s disease showed partly overlapping results (Fernández et al., 2013). The PD-MCI findings were only compared to those of PD-CN and healthy controls, which is not sufficient to determine if the reading impairments found are specific for PD-MCI or MCI in general. The inclusion of another MCI group, e.g., patients with MCI in early stage Alzheimer’s disease, may help to clarify the latter.

## Conclusion

The cognitive deficits that emerge in PD-MCI result in changes in reading pattern that are not restorable by levodopa, suggesting involvement of non- dopaminergic transmitter systems which is also assumed as the underlying pathophysiological concept of progressing cognitive decline in PD in general (Halliday et al., 2014). A longitudinal study is currently ongoing to identify eye movement patterns during reading as a potential surrogate marker of cognitive decline in PD.

More research is needed to clarify whether therapeutic interventions, e.g., medication, DBS, or neuropsychological training, provide a clinical benefit for PD patients with reading difficulties. Studying the effects of specific neuropsychological training of alternative reading strategies will be an interesting research field in the future.

## Data Availability Statement

The datasets generated for this study are available on request to the corresponding author.

## Ethics Statement

The studies involving human participants were reviewed and approved by Medizinische Fakultät Universität Marburg. The patients/participants provided their written informed consent to participate in this study.

## Author Contributions

LS contributed to organization and execution of research project and writing of the first draft. CK-Z and ZD contributed to execution of research project, review, and critique of draft. LT contributed to review and critique of draft. JW contributed to conception and organization of research project, statistical analysis, and writing of the first draft.

## Conflict of Interest

LT reports grants, personal fees, and non-financial support from SAPIENS Steering Brain Stimulation, Medtronic, Boston Scientific, and St. Jude Medical and has received payments from Bayer Healthcare, UCB Schwarz Pharma, and Archimedes Pharma and also honoraria as a speaker on symposia sponsored by Teva Pharma, Lundbeck Pharma, Bracco, Gianni PR, Medas Pharma, UCB Schwarz Pharma, Desitin Pharma, Boehringer Ingelheim, GSK, Eumecom, Orion Pharma, Medtronic, Boston Scientific, Cephalon, Abbott, GE Medical, Archimedes, and Bayer. JW reports financial support as a consultant for Boston Scientific. The remaining authors declare that the research was conducted in the absence of any commercial or financial relationships that could be construed as a potential conflict of interest.
